# The prevalence of obstructive sleep apnoea in women with polycystic ovary syndrome: a systematic review and meta-analysis

**DOI:** 10.1007/s11325-019-01835-1

**Published:** 2019-05-20

**Authors:** Hassan Kahal, Ioannis Kyrou, Olalekan A. Uthman, Anna Brown, Samantha Johnson, Peter D. H. Wall, Andrew Metcalfe, David G. Parr, Abd A. Tahrani, Harpal S. Randeva

**Affiliations:** 1 Division of Translational and Experimental Medicine, Warwick Medical School, University of Warwick, Coventry, CV4 7AL UK; 2 Warwickshire Institute for the Study of Diabetes, Endocrinology and Metabolism (WISDEM), University Hospitals Coventry and Warwickshire NHS Trust, Coventry, CV2 2DX UK; 3 Aston Medical Research Institute, Aston Medical School, Aston University, Birmingham, B4 7ET UK; 4 Centre of Applied Biological and Exercise Sciences (ABES), Faculty of Health and Life Sciences, Coventry University, Coventry, CV1 5FB UK; 5 Warwick - Centre for Applied Health Research and Delivery (WCAHRD), Division of Health Sciences, Warwick Medical School, University of Warwick, Coventry, CV4 7AL UK; 6 Library and Knowledge Services, University Hospitals Coventry and Warwickshire NHS Trust, Coventry, CV2 2DX UK; 7 University of Warwick Library, University of Warwick, Coventry, CV4 7AL UK; 8 Department of Warwick Orthopaedics, Warwick Medical School, University of Warwick, Coventry, CV2 2DX UK; 9 Department of Respiratory Medicine, Cardio-Respiratory Division, University Hospitals Coventry and Warwickshire NHS Trust, Coventry, CV2 2DX UK; 10 Institute of Metabolism and Systems Research, College of Medical and Dental Sciences, University of Birmingham, Birmingham, B15 2TT UK; 11 Centre of Endocrinology, Diabetes and Metabolism (CEDAM), Birmingham Health Partners, Birmingham, UK; 12 Department of Diabetes and Endocrinology, Birmingham Heartlands Hospital, Birmingham, UK

**Keywords:** PCOS, OSA, Hyperandrogenism, Obesity, Insulin resistance

## Abstract

**Background:**

Obesity is a common risk factor for polycystic ovary syndrome (PCOS) and obstructive sleep apnoea (OSA). Both PCOS and OSA are associated with increased risk of type 2 diabetes and cardiovascular disease. Hence, it is important to determine the burden of OSA in women with PCOS.

**Methods:**

We searched electronic databases (MEDLINE, Embase, CINAHL, PsycINFO, Scopus, Web of Science, OpenGrey, CENTRAL), conference abstracts, and reference lists of relevant articles, up to January 2019. No restriction for language or publication status. Studies that examined the presence of OSA in women with PCOS using polysomnography and/or level III devices were eligible for inclusion.

**Results:**

Seventeen studies involving 648 participants were included. Our meta-analysis showed that 35.0% (95% CI 22.2–48.9%) of women with PCOS had OSA. This prevalence was not affected by variation in PCOS definition between studies. Approximately one-tenth of the variation in OSA prevalence was related to differences in study population (higher in adults than adolescents and mixed populations), and around one-tenth was related to sample size (higher in smaller studies). OSA prevalence was markedly higher in obese versus lean women with PCOS, and in women with PCOS compared to controls (odds ratio = 3.83, 95% CI 1.43–10.24, eight studies, 957 participants (349 PCOS and 608 controls)). However, most of the studies were at high risk of selection bias, did not account for important confounders, included predominantly women with class II obesity, and were conducted in one country (USA).

**Conclusions:**

Future studies need to examine the true prevalence of OSA in a more representative sample of women with PCOS. Nevertheless, our results suggest that the prevalence of OSA in women with PCOS and obesity is high and clinicians should have a high index of suspicion of OSA in these women.

**Electronic supplementary material:**

The online version of this article (10.1007/s11325-019-01835-1) contains supplementary material, which is available to authorized users.

## Introduction

Polycystic ovary syndrome (PCOS) is the most frequent endocrine disorder in women of reproductive age, affecting around one in ten women [1–6]. The Rotterdam criteria [7] are widely used to diagnose PCOS, requiring the presence of two of the following features: (i) hyperandrogenism (clinical or biochemical), (ii) oligomenorrhoea/anovulation, and (iii) polycystic ovaries on ultrasound, and the exclusion of conditions with similar presentation [8]. PCOS carries a significant burden on the overall health of affected women as it is associated with multiple comorbidities, including obesity [9], subfertility [10], cardiovascular disease (CVD) risk [11], type 2 diabetes mellitus (T2DM) [12], depression, and impaired quality of life (QoL) [13–15]. Hyperandrogenism and obesity that are commonly seen in women with PCOS are believed to predispose these women to obstructive sleep apnoea (OSA) [16, 17].

OSA is a common disorder with data from a recent systematic review showing that the prevalence of OSA in women in the general population ranges from 6 to 19% [18]. OSA is characterised by recurrent episodes of partial or complete upper airway obstruction during sleep [19]. These episodes are associated with oxygen desaturations, sleep fragmentation, change in intra-thoracic pressure, sympathetic overactivity, and increase in heart rate and blood pressure [19, 20]. The risk of OSA increases with older age, obesity, male gender, smoking, alcohol intake, sedative use, menopause, and in certain ethnicities [19]. Similar to PCOS, OSA is a health burden in affected individuals and it is associated with increased risk of hypertension [21], CVD [22–24], mortality [25, 26], insulin resistance (IR) and T2DM [27], road traffic accidents [28], depression and impaired QoL [29–31], as well as subfertility [32].

In a recent systematic review and meta-analysis, OSA was associated with worse metabolic profile in women with PCOS [33]. However, despite its high prevalence and clinical implications, OSA often remains undiagnosed particularly in women [19, 34]. A few studies have suggested a high prevalence of OSA in women with PCOS [16, 35]. This is also supported by the Endocrine Society clinical practice guideline [36] which states in its evidence analysis that ‘women with PCOS develop OSA at rates that equal or exceed those in men’. However, the reported prevalence of OSA in women with PCOS has been very wide, ranging from 0 to 70% [17, 37], which raises concerns about the methodologies used in these studies. Similarly, a recent meta-analysis has attempted to answer this question [38]; however, the review contained significant methodological weaknesses that question its validity (‘OSA prevalence in PCOS’ in discussion). To date, there is no comprehensive, well-conducted, systematic review that examined the prevalence of OSA in women with PCOS. Assessing the prevalence of OSA in women with PCOS will allow the implementation of appropriate screening strategies and further development of clinical studies to examine the impact of OSA in women with PCOS. This is particularly important as OSA is a treatable condition, and it is associated with significant comorbidities [39].

## Objectives

To assess the prevalence of OSA in women with PCOS based on the existing literature.

## Methods

The protocol for this review was prospectively registered with PROSPERO: CRD42016041464 (http://www.crd.york.ac.uk/PROSPERO/) and is reported following the meta-analysis of Observational Studies in Epidemiology (MOOSE) guidelines [40].

### Selection criteria

Human studies, interventional or observational, that examined the presence of OSA in women with PCOS were included. We included studies that used polysomnography or level III devices to diagnose OSA, regardless of the cut-offs used. Conference abstracts and published studies were included.

All women with PCOS were included regardless of age (adults (premenopausal and postmenopausal) and adolescents (postmenarchal)), ethnicity, or PCOS diagnostic criteria.

Exclusion criteria are the following: conditions with presentation similar to PCOS including congenital adrenal hyperplasia, Cushing’s syndrome, prolactinomas, thyroid disease, and androgen-secreting tumours.

### Primary outcome

Prevalence of OSA in women with PCOS.

### Secondary outcomes

Prevalence of OSA in subpopulations of women with PCOS (obese, non-obese, adolescents, and adults).

### Search strategy

The initial literature search was performed on 11 April 2016 and updated on 7 February 2017, and on 11 January 2019. The search was not restricted by language or publication status. We searched Embase (Ovid), MEDLINE (Ebsco), PsycINFO (ProQuest), Scopus, CINAHL (Ebsco), OpenGrey, Web of Science, Cochrane Central Register of Controlled Trials (CENTRAL), and abstracts from major conferences. The reference lists of relevant papers and review articles were manually searched. The search strategy is provided in Appendix 1 (online data supplement).

### Selection of studies

Two authors (HK and IK) independently screened titles and abstracts. All potentially relevant articles were reviewed in full. Any disagreements between the two authors were resolved by consensus and, if necessary, discussion with a third author (OU).

### Data extraction and management

Two authors (HK and IK) independently extracted data. In case of multiple publications, we included the main study report/paper and, where necessary, additional details extracted from secondary papers. Study investigators were contacted to resolve any data queries, as required. For duplicate publications, we contacted study investigators to clarify the original publication, and if no response was received, we chose the study with the largest number of participants. We extracted the following details from each study: study design, participants’ characteristics, and prevalence estimates.

### Risk of bias

Two authors (HK and IK) independently assessed the risk of bias. Any disagreements between the two authors were resolved by consensus and discussion with a third author (OU), if necessary. We used the Risk of Bias Assessment Tool for Non-Randomised Studies (RoBANS) [41] to assess the risk of bias for included study. We assessed the following domains: selection bias (sample population), selection bias (confounding variables), performance bias (measurement of exposure), performance bias (analytical methods to control for bias), and other bias. The methodological components of the studies were assessed and classified as high risk, low risk, or unclear.

### Unit of analysis

Number of women with OSA in a population of women with PCOS.

### Data synthesis

We pooled the prevalence estimates using the DerSimonian–Laird random effects model [42]. We assessed the between study variations in prevalence estimates using the Higgins *I*^2^ statistic, and, as recommended, a value of greater than 50% was considered for moderate heterogeneity [43, 44]. We conducted series of meta-regression and subgroup analyses to explore the effect of study-level covariates on the prevalence estimates. The following study-level factors were considered: type of publication (conference abstract or journal article), year of publication (earlier studies: 2001 to 2009 or recent studies: 2010 to 2015), country, population (adults, adolescent, or mixed population), body mass index (non-obese versus obese), sample size, and PCOS definition (National Institutes of Health (NIH) definition [45], Rotterdam criteria [7], or not reported). The possibility of reporting bias was assessed using an Egger’s test and funnel plot [46]. Leave-one-study-out sensitivity analysis was also performed to assess the stability of the meta-analysis result [47]. Stata 14 was used for all analyses (StataCorp, College Station, TX).

## Results

### Search results and study characteristics

**Fig. 1:**
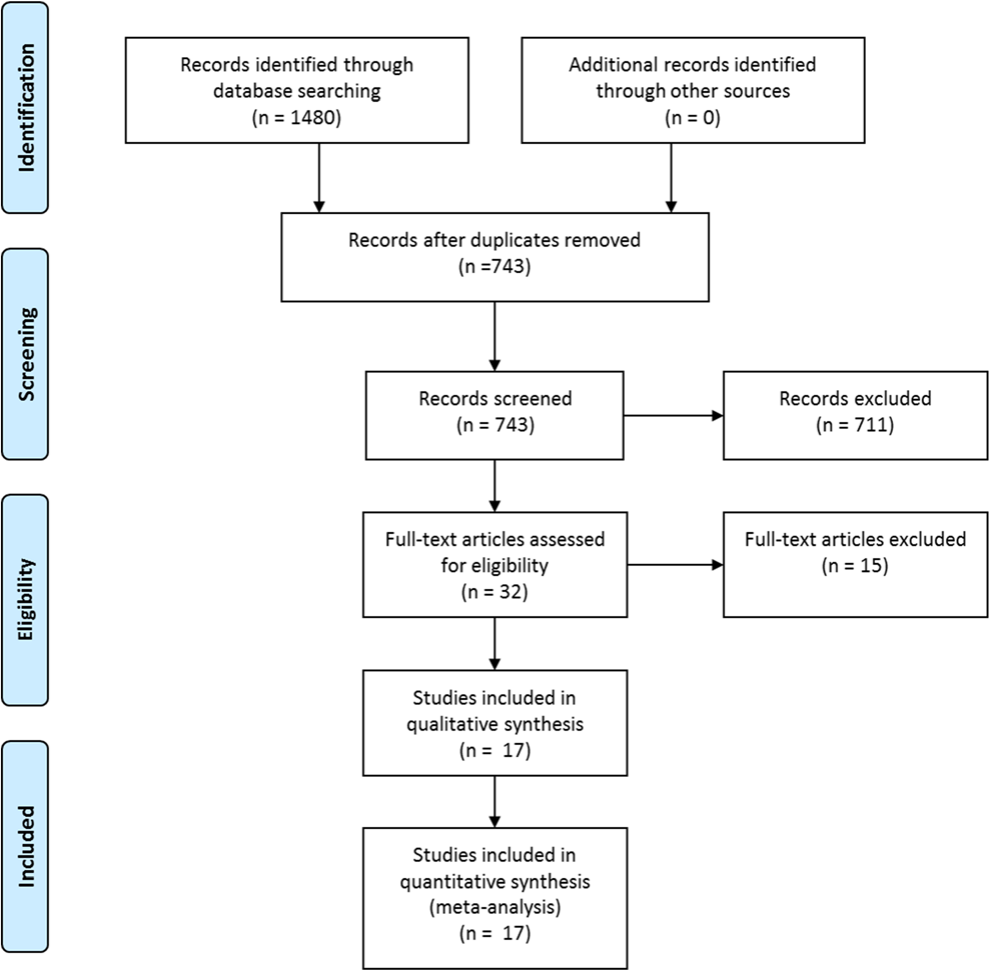
PRISMA flow diagram

The search results are shown in a PRISMA flow diagram (Fig. 1). Main characteristics of the 17 included and 15 excluded studies are summarised in Table 1 and Table S1 (in the online data supplement), respectively. All of the included studies used polysomnography to diagnose OSA and were reported in English. Five studies did not specify the cut-offs used to diagnose OSA; three studies used an apnoea/hypopnoea index (AHI) > 5 events per hour, and nine studies used variable diagnostic criteria (Table 1).

**Table 1:** Characteristics of included studies [ordered alphabetically based on the first author’s surname]

Study	Country	Study design	Publication type	Population	*N*	Ethnicity	Age	BMI	PCOS diagnosis	OSA diagnosis	
							(yr)	(kg/m^2^)	Criteria	Method	Events/h
Abdel Wahab 2013 [48]	Egypt	CS	C. abstract	Adults	21	NR	32.5 ± 6.3	38.1 ± 11.7	NR	PSG	NR
Chatterjee 2014 [49]	India	CS	J. article	Adults	50	South Asian	NR	28 ± 3.0	Rotterdam	PSG	RDI ≥ 5 + symptoms or RDI > 15
de Sousa 2012 [50]	Germany	CS	J. article	Adolescents	35	NR	15.2 ± 1.0	33.2 ± 6.8	NIH	PSG	NR
Fogel 2001 [35]	USA	CS	J. article	Adults	18	NR	31.1 ± 5.5	36.9 ± 5.5	NIH	PSG	AHI > 5
Gateva 2013 [51]	Bulgaria	CS	C. abstract	Adults	19	NR	NR	NR	NR	PSG	NR
Gopal 2002 [17]	USA	CS	J. article	Adults	23	NR	NR	42.7 ± 8.5	NR	PSG	RDI ≥ 5 + symptoms
Kenigsberg 2015 [52]	USA	CS	C. abstract	Mixed (13–21 yr)	31	NR	16.7 ± 2.4	NR	Rotterdam	PSG	AHI > 2
Morselli 2013 [53]	USA	CS	C. abstract	Adults	21	AA	28 ± 1.0	39.0 ± 2.0	NR	PSG	AHI > 5
Nandalike 2012 [54]	USA	CS	J. article	Adolescents	28	17.9% AA, 14.3% Hispanic, 14.3% White, 53.6% mixed	16.8 ± 1.9	44.8 ± 8.8	Rotterdam**	PSG	AHI > 5 or AI > 1
Saha 2018 [55]	India	CS	C. abstract	Adults	64*	South Asian	24.3 ± 4	26.4 ± 4.4	Rotterdam	PSG	AHI ≥ 5
Tasali 2008 [56]	USA	CS	J. article	Adults	52	62% AA or Hispanic	29.7 ± 5.1	39.2 ± 7.2	NIH	PSG	AHI ≥ 5
Temple 2013 [57]	USA	CS	C. abstract	Adults	129	NR	28.2 ± 5.7	38.6 ± 6.8	NR	PSG	NR
Tock 2014 [58]	Brazil	CS	J. article	Mixed (16–45 yr)	38	NR	28.3 ± 6.8	32.9 ± 7.7	Rotterdam	PSG	AHI ≥ 5
Vgontzas 2001 [16]	USA	CS	J. article	Mixed (16–45 yr)	53	NR	30.4 ± 6.6	38.7 ± 8.0	NIH	PSG	AHI ≥ 10 + symptoms
Wootton 2017 [59]	USA	CS	C. abstract	Adolescents	16	NR	NR	NR	Rotterdam	PSG	AHI > 5 or AI > 1, and SpO2 < 90%
Yang 2009 [37]	Taiwan	CS	J. article	Adults	18	Chinese	29.1 ± 6.1	21.7 ± 2.4	Rotterdam**	PSG	AHI ≥ 5
Zea-Hernandez 2014 [60]	USA	CS	C. abstract	Adolescents	32	NR	16.5 ± 1.6	36.1 ± 9.2	NR	PSG	NR

Data presented as mean ± standard deviation

*AA* African American, *AHI* apnoea hypopnoea index, *AI* apnoea index, *BMI* body mass index, *C. abstract* conference abstract, *CS* cross-sectional study, *h* hour, *J. article* journal article, *N* sample size, *NIH* National Institutes of Health, *NR* not reported, *OSA* obstructive sleep apnoea, *PCOS* polycystic ovary syndrome, *PSG* polysomnography, *RDI* respiratory disturbance index, *yr* years

*Total sample size was confirmed with authors

**All participants also fulfilled NIH criteria

### Risk of bias of included studies

**Fig. 2:**
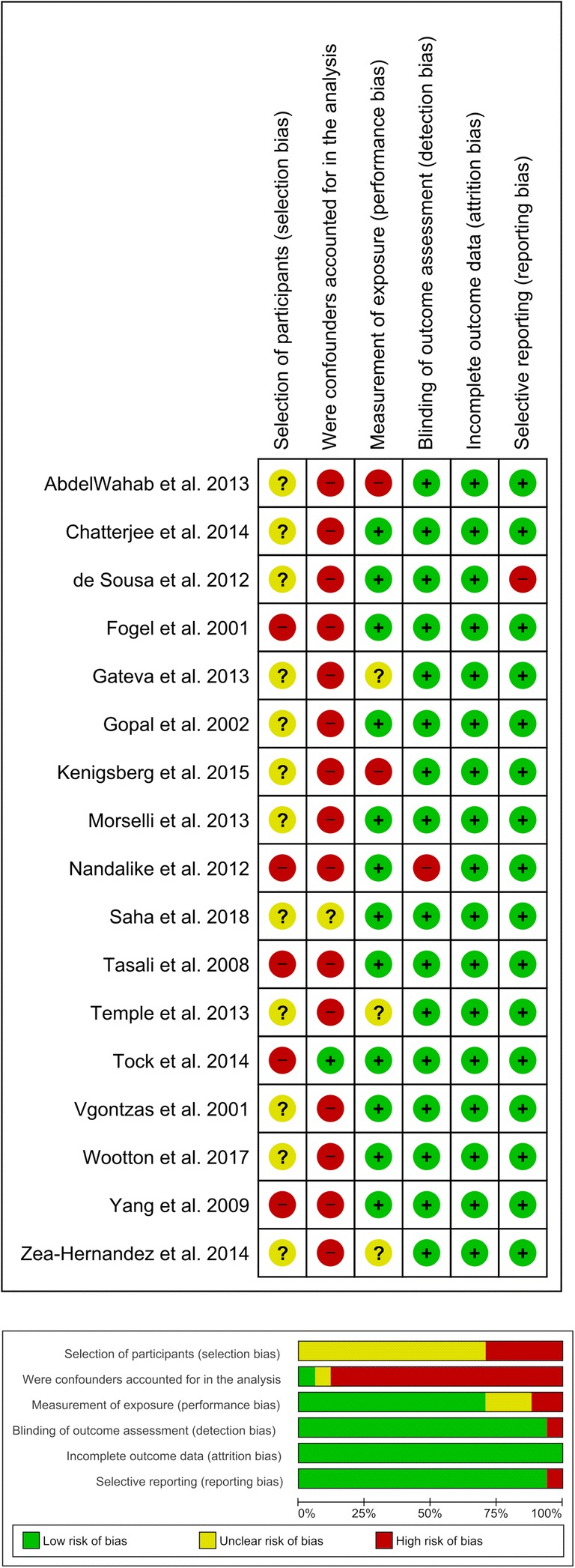
Risk of bias of included studies

The risk of bias assessment summary for each study is presented in Fig. 2. The selection bias due to inadequate selection of participants was high in five studies and unclear in the remaining 12 studies. The selection bias caused by the inadequate confirmation and consideration of confounding variables was high in most of the studies (15 out of 17 studies) and low only in one study. The performance bias due to inadequate measurement of exposure was low in 12 studies, high in two studies, and unclear in the remaining three studies. The detection bias due to inadequate blinding of outcome assessments was low in most studies (16 out of 17 studies) and high only in one study. The attrition bias due to inadequate handling of incomplete outcome data was low in all 17 studies. The reporting bias due to selective reporting was low in most studies (16 out of 17 studies) and high in one study.

### Overall prevalence of OSA in women with PCOS

**Fig. 3:**
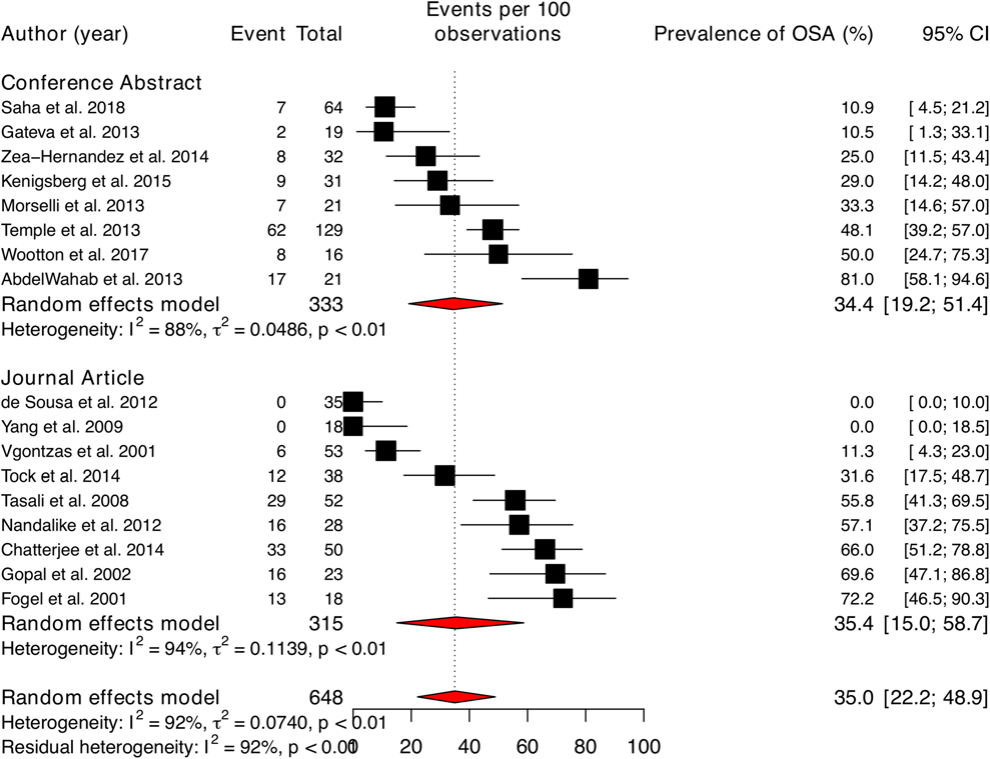
Pooled prevalence of obstructive sleep apnoea (OSA)

The prevalence of OSA in women with PCOS and 95% CIs from individual studies with a pooled estimate are shown in Fig. 3. The pooled prevalence of OSA for all studies yielded an estimate of 35.0% (95% CI 22.2 to 48.9%) of women with PCOS. The *I*^2^ statistic was 92%, indicating statistically significant heterogeneity among the studies. The contour-enhanced funnel plot of examination of publication bias is shown in Fig. S1 in the online data supplement. We found no evidence of publication bias as indicated by the relatively symmetrical funnel plot of studies’ precision against prevalence estimates (in logarithmic scale). This was confirmed when formally tested using the Egger method (*p* value for small study bias = 0.063). The results of leave-one-study-out sensitivity analyses showed that no study had undue influence on the pooled OSA prevalence; see Fig. S2 in the online data supplement.

### Prevalence of OSA by different subgroups

**Fig. 4:**
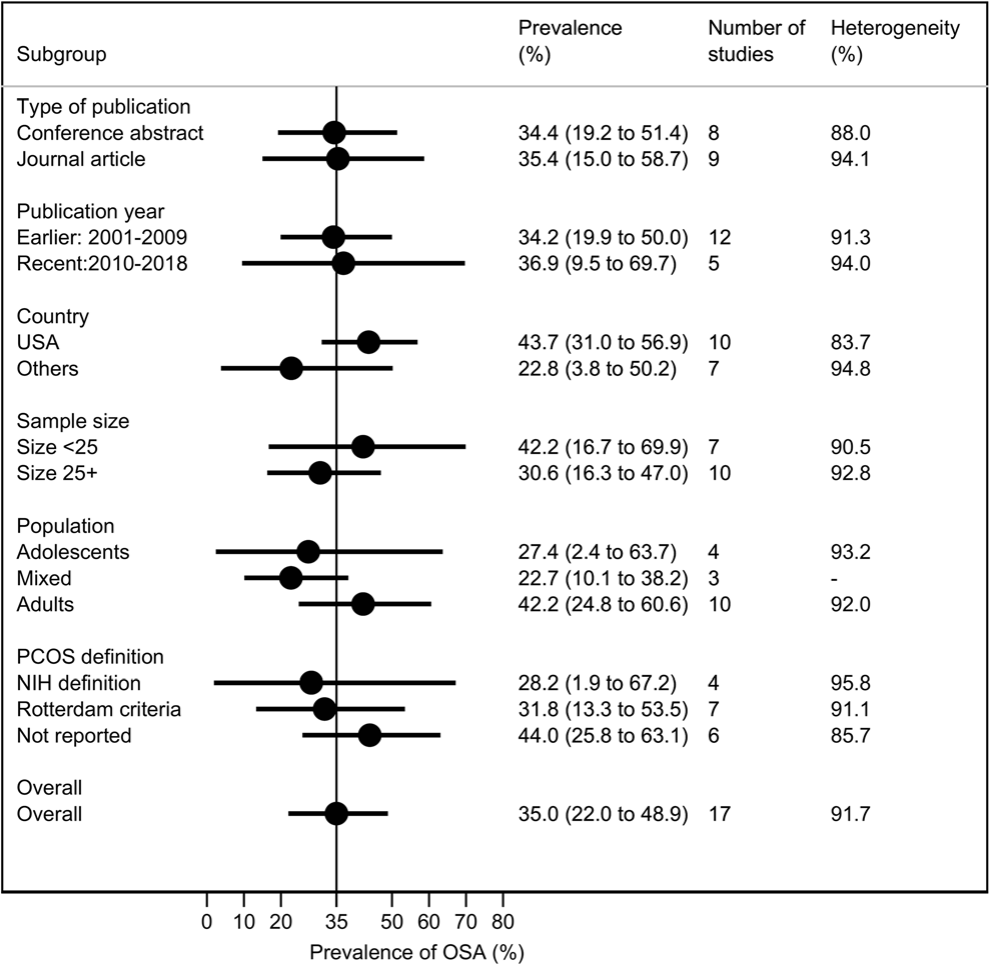
Pooled prevalence of obstructive sleep apnoea (OSA) by different subgroup

**Fig. 5:**
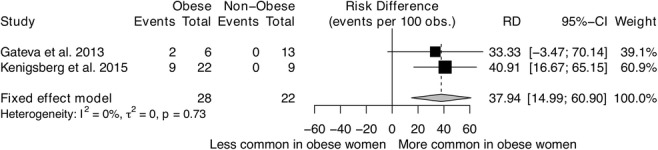
Obstructive sleep apnoea (OSA) prevalence difference in women with PCOS and obesity versus women with PCOS without obesity. In the study by Gateva et al., the cut-offs used to define obesity in women with PCOS were not reported. Kenigsberg et al. included females with PCOS between the ages of 13–21 years and defined obesity as a BMI *z*-score > 95th percentile, while participants with BMI *z*-score < 85th percentile were considered lean

The results of subgroup analyses are shown in Fig. 4. There is no evidence of statistically significant difference in the pooled prevalence of OSA by type of publication, publication year, or sample size. Studies published in the USA tended to report higher prevalence of OSA than studies from other countries (43.7% versus 22.8%); however, this difference did not reach a statistically significant level (*p* = 0.725). Compared to studies that used the NIH PCOS definition, studies that used the Rotterdam criteria and those not reporting how PCOS was diagnosed tended to have higher OSA prevalence, albeit not statistically significant. Two studies stratified the prevalence estimates by body mass index, i.e. non-obese versus obese women (Fig. 5). For both studies, the prevalence of OSA was 0% in the non-obese population. In the Gateva and colleagues study [51], two of the six obese women had OSA (33%), while in the Kenigsberg and colleagues study [52], nine out of 22 obese women had OSA (41%). When these two studies were pooled, the prevalence of OSA was 38% higher in women with obesity and PCOS compared to non-obese women with PCOS (prevalence difference = + 37.9%, 95% CI 15.0 to 60.9%).

### Factors modifying the prevalence of OSA as identified by meta-regression analysis

Factors associated with prevalence estimates and proportion of explained variability in prevalence estimates as identified by meta-regression analyses are shown in Table S2 in the online data supplement. In a series of meta-regression analyses, none of the study-level factors were significantly associated with the prevalence estimates. However, approximately one-tenth of the between study variation in the prevalence estimates was explained by the variations in the study sample size (higher in smaller studies), and around one-tenth was related to differences in the study population (higher in adults compared to adolescents and mixed populations).

## Discussion

Our analysis of the existing data suggests that OSA prevalence in women with PCOS is high. This is mainly driven by studies that were conducted in the USA, included women with PCOS and class II obesity, and had a relatively high level of selection bias. Accordingly, it is difficult to draw firm conclusions about the true prevalence of OSA in women with PCOS based on the available data. As expected, the prevalence of OSA in women with PCOS appears to increase with age and obesity.

### OSA prevalence in PCOS

Based on our meta-analysis, OSA is present in almost one-third of women with PCOS. In the recent meta-analysis by Helvaci et al., the prevalence of OSA was lower at 0.22 (95% CI 0.08–0.40) in women with PCOS [38]. The lower OSA prevalence reported could be explained by differences in study methodology. In the study by Helvaci et al., the literature search was limited to two electronic databases, was restricted to studies published in English language, and did not include ‘grey literature’ (for example, conference abstracts). In addition, in their meta-analysis, Helvaci et al. included duplicate publications [50, 61–63], and one study with 0% prevalence of OSA as only women without OSA were recruited [64].

**Fig. 6:**
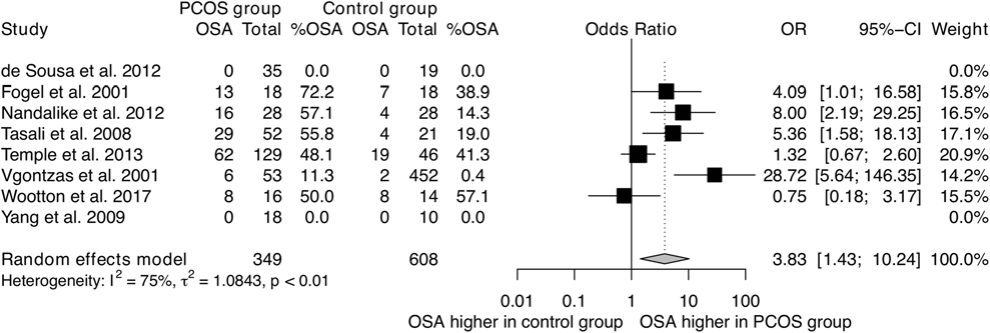
The prevalence of obstructive sleep apnoea in women with PCOS compared to controls

The prevalence of OSA in women in the general population has been reported at 6–19% [18]. Seven studies have compared OSA prevalence in women with PCOS to matched controls, and the majority [16, 35, 54, 56, 57] have shown OSA to be more prevalent in women with PCOS (Table 2). Women with PCOS were four times more likely to have developed OSA than controls (odds ratio = 3.83, 95% CI 1.43–10.24, eight studies, 957 participants) (Fig. 6). However, important confounding factors that are known to affect the risk of OSA have not been adequately considered in these studies, including obesity [16], abdominal adiposity [16, 35, 54, 56], and ethnicity [16, 54]. Moreover, while controls were recruited from the general population [16, 35, 56], women with PCOS were recruited from specialised clinics [35, 37, 56]. In addition, PCOS was not formally ruled out in controls [16]. Subsequently, it is not clear if women with PCOS in the community are at higher risk of OSA compared to age- and adiposity-matched controls. Well-conducted large observational studies in the general population are needed to answer this question.

**Table 2:** Characteristics of studies comparing obstructive sleep apnoea (OSA) prevalence in women with polycystic ovary syndrome (PCOS) to controls [ordered alphabetically based on the first author’s surname]

Study	Country	Population	OSA	PCOS				Controls				P (OSA %)	Notes
			Diagnosis method	*N*	Age (yr)	BMI (kg/m^2^)	OSA (%)	*N*	Age (yr)	BMI (kg/m^2^)	OSA (%)	PCOS versus controls	
de Sousa 2012 [50]	Germany	Adolescents	PSG	35	15.2 ± 1.0	33.2 ± 6.8	0.0%	19	15.2 ± 1.1	32.4 ± 3.9	0.0%	NS	HOMA-IR and androgen levels were higher in subgroups of girls with POCS than controls.
Fogel 2001 [35]	USA	Adults	PSG	18	31.1 ± 5.5	36.9 ± 5.5	72%	18	32.3 ± 5.5	36.9 ± 5.9	39%	0.10	Women with PCOS had higher waist-to-hip ratio and testosterone levels.
Nandalike 2012 [54]	USA	Adolescents	PSG	28	16.8 ± 1.9	44.8 ± 8.8	57.1%	28	17.1 ± 1.8	40.2 ± 4.7	14.3%	< 0.01	Only girls with symptoms were tested. No difference in BMI *z*-scores between groups. More girls in the PCOS group (32%) had a history of adenotonsillectomy (treatment of choice for OSA in children) prior to PSG compared controls (10.7%).
Tasali 2008 [56]	USA	Adults	PSG	52	29.7 ± 5.1	39.2 ± 7.2	55.8%	21	30.7 ± 5.0	36.0 ± 6.9	19%	0.01	The authors adjusted for BMI and ethnicity in their analysis of OSA prevalence.
Temple 2013 [57]	USA	Adults	PSG	129	28.2 ± 5.7	38.6 ± 6.8	48.1%	46	31.0 ± 6.1	39.4 ± 7.5	41%	0.03	The authors adjusted for age, BMI, and race in their analysis of OSA prevalence.
Vgontzas 2001 [16]	USA	Mixed (16–45 yr)	PSG	53	30.4 ± 6.6	38.7 ± 8.0	11.3%	452	32.1 ± 6.4	26.4 ± 6.4	0.4%	< 0.0001	Despite the authors adjusting for BMI, statistical adjustment is unlikely to fully exclude an obesity-related effect on OSA prevalence as the difference in BMI was high (12.3 kg/m^2^).
Wootton 2017 [59]	USA	Adolescents	PSG	16	NR	NR	50%	14	NR	NR	57.1%	NS	Total *N* = 30 girls, age 14–18 years; and mean BMI 37.2 kg/m^2^ (range 22–55 kg/m^2^). No information on how participants were selected, or differences between subgroups in age, BMI, etc.
Yang 2009 [37]	Taiwan	Adults	PSG	18	29.1 ± 6.1	21.7 ± 2.4	0.0%	10	31.6 ± 12.3	20.9 ± 1.8	0.0%	NS	PCOS group had higher testosterone levels and larger waist circumference and WHR compared to controls.

Data presented as mean ± standard deviation

*BMI* body mass index, *HOMA-IR* homeostatic model assessment of insulin resistance, *WHR* waist-to-hip ratio, *N* sample size, *NR* not reported, *NS* not significant, *OSA* obstructive sleep apnoea, *OSA %* OSA prevalence, *PCOS* polycystic ovary syndrome, *PSG* polysomnography, *yr* years

### Factors affecting OSA prevalence in PCOS

We found a trend for OSA to be more common in adult women with PCOS compared to adolescents, and in women with PCOS and obesity compared to women with PCOS without obesity. This is not surprising as the risk of OSA in the general population increases with increasing age and obesity [19, 65]. Therefore, it is likely that PCOS precedes the development of OSA, particularly as features of PCOS have been identified in prepubertal girls [66, 67]. However, it is also possible that OSA may precede the development of PCOS in some women [68]; for example, a third of the adolescent girls with PCOS in the study by Nandalike et al. [54] had a previous history of tonsillectomy, and tonsillitis and/or tonsillar enlargement are the most common cause of OSA in children [69].

The limited data available suggest that OSA prevalence is low in women with PCOS who are lean/non-obese. This is based on three studies that reported data on a total of 40 participants with these characteristics [37, 51, 52]. However, the sample size is too small to draw firm conclusions.

Androgens are believed to play a role in the pathogenesis of OSA and may contribute to the higher prevalence of OSA in men compared to women [19]. However, despite the higher levels of testosterone and/or free testosterone in the PCOS group compared to controls in three studies [37, 50, 53], there was no difference in OSA prevalence (Table 2). Furthermore, while some studies showed the severity of OSA in women with PCOS to correlate with hyperandrogenism [35]; others did not show a similar relationship [56]. In addition, while women with PCOS diagnosed based on the NIH definition are expected to show higher degree of hyperandrogenism compared to those diagnosed using the Rotterdam criteria, we did not find a significant difference in OSA prevalence based on the PCOS diagnostic criteria/definition used. Subsequently, based on the existing data, the role of hyperandrogenism in influencing OSA risk in women with PCOS is probably small, which might be due to the relatively lower levels of androgens in women with PCOS compared to men.

Large cohort studies with long duration of follow-up are needed to clarify the natural history and relationship between PCOS, OSA, and obesity.

### PCOS and OSA... chicken-and-egg?

While obesity is a shared feature between PCOS and OSA that may contribute to the high prevalence of OSA in women with PCOS reported in some studies, other factors may also play an important role in how these two conditions interact [68].

PCOS may contribute to the pathogenesis of OSA through its association with hyperandrogenism and low progesterone levels and may lead to increased upper airway collapsibility, and/or changes in the sensitivity and responsiveness of the ventilatory chemoreceptors [70].

On the other hand, OSA may contribute to the pathogenesis of PCOS through promoting the following: (i) IR [71]; (ii) oxidative stress [72], which may exacerbate IR [73] and contribute to infertility through disruption of the meiotic spindle formation in the oocyte [74]; and (iii) increased sympathetic activity that may lead to IR, altered ovarian function, and the development polycystic ovarian morphology [75, 76].

Subsequently, it is plausible that the relationship between PCOS and OSA is bidirectional [33, 68] (Fig. S3 in the online data supplement). Understanding this relationship is important, as many of the mechanisms through which OSA might impact on PCOS (e.g. IR, inflammation [77], oxidative stress, and sympathetic activation) are amenable to treatment with continuous positive airway pressure (CPAP) [78, 79]. In addition, due to the increased risk of road traffic accidents in patients with OSA, it is important to identify these patients as CPAP lowers this risk [80]. Well-conduced longitudinal large cohort studies and randomised controlled trials are needed to assess the exact relationship between these two common conditions.

## Study limitations

We found high heterogeneity in the reported studies. However, this is not unexpected in observational studies. We conducted meta-regression analyses to explore between study-level factors that could explain the observed between study heterogeneity and found that the study population (i.e. adolescents, adults, mixed), and study sample size each explained around one-tenth of the noted heterogeneity. In addition, to prevent wrong or misleading conclusions, meta-analysis rather than narrative synthesis has been recommended even in the presence of high between study variations in the prevalence estimates. Examining the effects of the different cut-offs used to diagnose OSA on its prevalence was not possible as, while some studies reported AHI, others reported RDI or AI when diagnosing OSA, and five studies did not report how OSA was diagnosed. The majority of studies that examined OSA prevalence in PCOS was found to be at high risk of selection bias and did not account for important confounding factors. Subsequently, the prevalence of OSA in women with PCOS reported in this systematic review should be viewed with some caution.

We would like to note a few deviations from our original protocol: we included studies that used level III devices, in addition to polysomnography, as they are validated and approved methods to diagnose OSA [81]. However, all the studies we identified have used polysomnography to diagnose OSA. We accepted the study authors’ own definitions of OSA and PCOS, regardless of the diagnostic criteria used. In five conference abstracts and two journal articles, the authors did not report the criteria used to diagnose OSA and/or PCOS (Table 1). However, we do not believe that these deviations affected our results since we did not find a significant difference in OSA prevalence between conference abstracts and journal articles, neither did we find a difference in OSA prevalence based on the PCOS diagnostic criteria used. In addition, the AHI threshold to diagnose OSA differs between children and adults.

## Study strengths

We conducted and reported this systematic review and meta-analysis following internationally recognised recommendations. We have searched a large number of databases/sources to identify relevant articles, and our search was not restricted by study design, language, or publication type or year.

## Conclusions

Obstructive sleep apnoea appears to affect a third of women with polycystic ovary syndrome, but this is mainly driven by studies that recruited women with class II obesity and had significant limitations. Whether, and to what extent, women with PCOS are at increased risk of OSA, compared to women without PCOS, remains unclear. Well-conducted, large, cohort studies in the general population with extended follow-up are required to assess the true prevalence of OSA in women with PCOS and to examine the natural history and relationship between PCOS, OSA, and obesity.

## Electronic supplementary material

ESM 1 (DOCX 672 kb)
