# Supplementary material for: The prevalence of obstructive sleep apnoea in women with polycystic ovary syndrome: a systematic review and meta-analysis
Source: Sleep Breath. 2019 May 20;24(1):339–50. doi: 10.1007/s11325-019-01835-1 (PMC7127997; doi:10.1007/s11325-019-01835-1)
Supplement: Supplementary file 1 — (DOCX 672 kb) [file 11325_2019_1835_MOESM1_ESM.docx]

**Online data supplement**

**Title: The Prevalence of Obstructive Sleep Apnoea in women with Polycystic Ovary Syndrome: a Systematic Review and Meta-analysis.**

Hassan Kahal^1,2^, Ioannis Kyrou^1,2,3,4^, Olalekan A Uthman^5^, Anna Brown^6^, Samantha Johnson^7^, Peter D. H. Wall^8^, Andrew Metcalfe^8^, David G Parr^9^, Abd A Tahrani*^10,11,12^, Harpal S Randeva*^1,2,3,4^

^1^Division of Translational and Experimental Medicine, Warwick Medical School, University of Warwick, Coventry, CV4 7AL, United Kingdom.

^2^Warwickshire Institute for the Study of Diabetes, Endocrinology and Metabolism (WISDEM), University Hospitals Coventry and Warwickshire NHS Trust, Coventry CV2 2DX, United Kingdom.

^3^Aston Medical Research Institute, Aston Medical School, Aston University, Birmingham, B4 7ET, United Kingdom.

^4^Centre of Applied Biological & Exercise Sciences (ABES), Faculty of Health & Life Sciences, Coventry University, Coventry, CV1 5FB, UK.

^5^Warwick - Centre for Applied Health Research and Delivery (WCAHRD), Division of Health Sciences, Warwick Medical School, University of Warwick, Coventry, CV4 7AL, United Kingdom.

^6^Library and Knowledge Services, University Hospitals Coventry and Warwickshire NHS Trust, Coventry, CV2 2DX, United Kingdom.

^7^University of Warwick Library, University of Warwick, Coventry, CV4 7AL, United Kingdom.

^8^Department of Warwick Orthopaedics, Warwick Medical School, University of Warwick, Coventry, CV2 2DX, United Kingdom.

^9^Department of Respiratory Medicine, Cardio-Respiratory Division, University Hospitals Coventry and Warwickshire NHS Trust, Coventry CV2 2DX, United Kingdom.

^10^Institute of Metabolism and Systems Research, College of Medical and Dental Sciences, University of Birmingham, Birmingham, B15 2TT, United Kingdom.

^11^Centre of Endocrinology, Diabetes and Metabolism (CEDAM), Birmingham Health Partners, Birmingham, UK

^12^Department of Diabetes and Endocrinology, Birmingham Heartlands Hospital, Birmingham, UK.

*Joint senior authors; contributed equally to the manuscript.

**Short title:** OSA prevalence in PCOS

**Corresponding Author:** Professor Harpal Randeva. Warwickshire Institute for the Study of Diabetes, Endocrinology and Metabolism (WISDEM), University Hospitals Coventry and Warwickshire NHS Trust, Coventry CV2 2DX, Email: [Harpal.Randeva@warwick.ac.uk](mailto:Harpal.Randeva@warwick.ac.uk).

**Table S1 Characteristics of excluded studies *[ordered by year of study]*.**

| **Study** | **Reason for exclusion** |
| --- | --- |
| Tasali 2006 [1] | No information on how many women had obstructive sleep apnoea |
| Vgontzas 2006 [2] | Only participants without obstructive sleep apnoea were included |
| De Sousa 2010 [3] | Duplicate publication to De Sousa 2012 [4] |
| De Sousa 2010 [5] | Duplicate publication to De Sousa 2012 [4] |
| Yang 2010 [6] | Duplicate publication to Yang 2009 [7] |
| De Sousa 2011 [8] | Duplicate publication to De Sousa 2012 [4] |
| De Sousa 2011 [9] | Duplicate publication to De Sousa 2012 [4] |
| Nandalike 2011 [10] | No sleep studies performed |
| Tasali 2011 [11] | Only women with obstructive sleep apnoea were included |
| De Sousa 2012 [12] | Duplicate publication to De Sousa 2012 |
| Mokhlesi 2012 [13] | No sleep studies performed |
| Temple 2013 [14] | Duplicate publication to Temple 2013 [15] |
| Suri 2016 [16] | Duplicate publication to Chatterjee 2014 [17] |
| Hachul 2017 [18] | The prevalence of OSA was not reported |
| Kahal 2018 [19] | Women with low risk of OSA did not have sleep studies performed |

**References:**

1. Tasali E, Van Cauter E, Ehrmann DA (2006) Relationships between sleep disordered breathing and glucose metabolism in polycystic ovary syndrome. J Clin Endocrinol Metab 91 (1):36-42. doi:10.1210/jc.2005-1084

2. Vgontzas AN, Trakada G, Bixler EO, Lin HM, Pejovic S, Zoumakis E, Chrousos GP, Legro RS (2006) Plasma interleukin 6 levels are elevated in polycystic ovary syndrome independently of obesity or sleep apnea. Metabolism 55 (8):1076-1082. doi:10.1016/j.metabol.2006.04.002

3. de Sousa G, Schluter B, Buschatz D, Menke T, Trowitzsch E, Andler W, Reinehr T (2010) A comparison of polysomnographic variables between obese adolescents with polycystic ovarian syndrome and healthy, normal-weight and obese adolescents. Sleep Breath 14 (1):33-38. doi:10.1007/s11325-009-0276-0

4. de Sousa G, Schluter B, Buschatz D, Menke T, Trowitzsch E, Andler W, Reinehr T (2012) The impact of insulin resistance and hyperandrogenemia on polysomnographic variables in obese adolescents with polycystic ovarian syndrome. Sleep Breath 16 (1):169-175. doi:10.1007/s11325-010-0469-6

5. de Sousa G, Schlüter B, Menke T, Trowitzsch E, Andler W, Reinehr T (2010) A Comparison of Polysomnographic Variables between Adolescents with Polycystic Ovarian Syndrome and Healthy Controls. Int J Clinical Medicine 1:48-53. doi:10.4236/ijcm.2010.12009

6. Yang HP, Kang JH, Su HY, Huang SY (2010) A pilot study of heart rate variability and apneic-hypopneic events in non-obese women with polycystic ovary syndrome during sleep. Nutr Sci J 35 (1):9-21

7. Yang HP, Kang JH, Su HY, Tzeng CR, Liu WM, Huang SY (2009) Apnea-hypopnea index in nonobese women with polycystic ovary syndrome. Int J Gynaecol Obstet 105 (3):226-229. doi:10.1016/j.ijgo.2009.02.004

8. de Sousa G, Schluter B, Menke T, Trowitzsch E, Andler W, Reinehr T (2011) Relationships between polysomnographic variables, parameters of glucose metabolism, and serum androgens in obese adolescents with polycystic ovarian syndrome. J Sleep Res 20 (3):472-478. doi:10.1111/j.1365-2869.2010.00902.x

9. de Sousa G, Schluter B, Menke T, Trowitzsch E, Andler W, Reinehr T (2011) A comparison of polysomnographic variables between adolescents with polycystic ovarian syndrome with and without the metabolic syndrome. Metab Syndr Relat Disord 9 (3):191-196. doi:10.1089/met.2010.0081

10. Nandalike K, Strauss T, Agarwal C, Coupey SM, Sin S, Rajpathak S, Cohen HW, Arens R (2011) Screening for sleep-disordered breathing and excessive daytime sleepiness in adolescent girls with polycystic ovarian syndrome. J Pediatr 159 (4):591-596. doi:10.1016/j.jpeds.2011.04.027

11. Tasali E, Chapotot F, Leproult R, Whitmore H, Ehrmann DA (2011) Treatment of obstructive sleep apnea improves cardiometabolic function in young obese women with polycystic ovary syndrome. J Clin Endocrinol Metab 96 (2):365-374. doi:10.1210/jc.2010-1187

12. de Sousa G, Schluter B, Menke T, Trowitzsch E, Andler W, Reinehr T (2012) Longitudinal analyses of polysomnographic variables, serum androgens, and parameters of glucose metabolism in obese adolescents with polycystic ovarian syndrome. Sleep Breath 16 (4):1139-1146. doi:10.1007/s11325-011-0620-z

13. Mokhlesi B, Scoccia B, Mazzone T, Sam S (2012) Risk of obstructive sleep apnea in obese and nonobese women with polycystic ovary syndrome and healthy reproductively normal women. Fertil Steril 97 (3):786-791. doi:10.1016/j.fertnstert.2011.12.024

14. Temple KA, Leproult R, Whitmore H, Mokhlesi B, Van Cauter E, Ehrmann DA (2013) Adiponectin levels in obese women with and without PCOS: Impact of obstructive sleep apnea. Paper presented at the 95th Annual Meeting and Expo of the Endocrine Society, ENDO, San Francisco, CA United States,

15. Temple KA, Tasali E, Mokhlesi B, Whitmore H, Watson S, Van Cauter E, Ehrmann DA (2013) Abnormal glucose tolerance in women with polycystic ovary syndrome (PCOS): Role of sex steroids and obstructive sleep apnea. Paper presented at the 73rd Scientific Sessions of the American Diabetes Association, Chicago, IL United States,

16. Suri J, Suri JC, Chatterjee B, Mittal P, Adhikari T (2016) Obesity may be the common pathway for sleep-disordered breathing in women with polycystic ovary syndrome. Sleep Med 24:32-39. doi:10.1016/j.sleep.2016.02.014

17. Chatterjee B, Suri J, Suri JC, Mittal P, Adhikari T (2014) Impact of sleep-disordered breathing on metabolic dysfunctions in patients with polycystic ovary syndrome. Sleep Med 15 (12):1547-1553. doi:10.1016/j.sleep.2014.06.023

18. Hachul H, Polesel D, Tock L, Carneiro G, Pereira A, Zanella MT, Tufik S, Togeiro SM (2017) Sleep disorders in women with polycystic ovary syndrome: The influence of obesity and hyperandrogenism. Sleep Medicine 40 (Supplement 1):e125

19. Kahal H, Tahrani AA, Kyrou I, Dimitriadis GK, Kimani PP, Barber TM, Nicholls M, Ali A, Weickert MO, Randeva HS (2018) The relationship between obstructive sleep apnoea and quality of life in women with polycystic ovary syndrome: a cross-sectional study. In: Endocrine Abstracts. Society for Endocrinology BES 2018. Bioscientifica,

**Figure S1 Funnel plot for publication bias.**

**
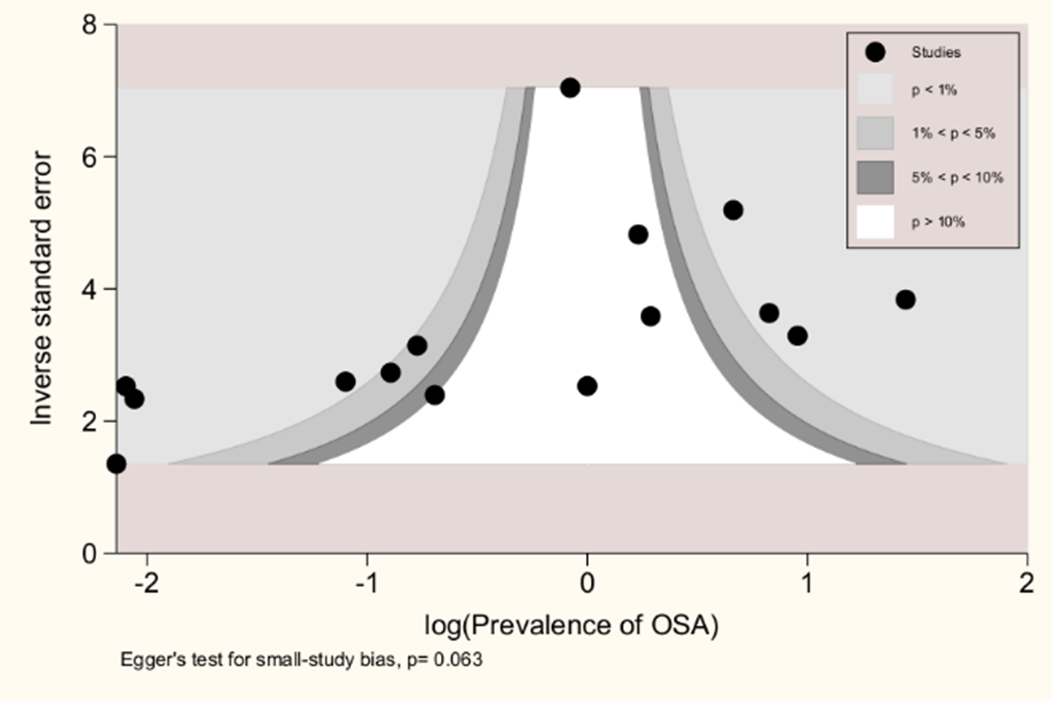
**

**Figure S2 Leave-one-out sensitivity analyses of obstructive sleep apnoea prevalence**


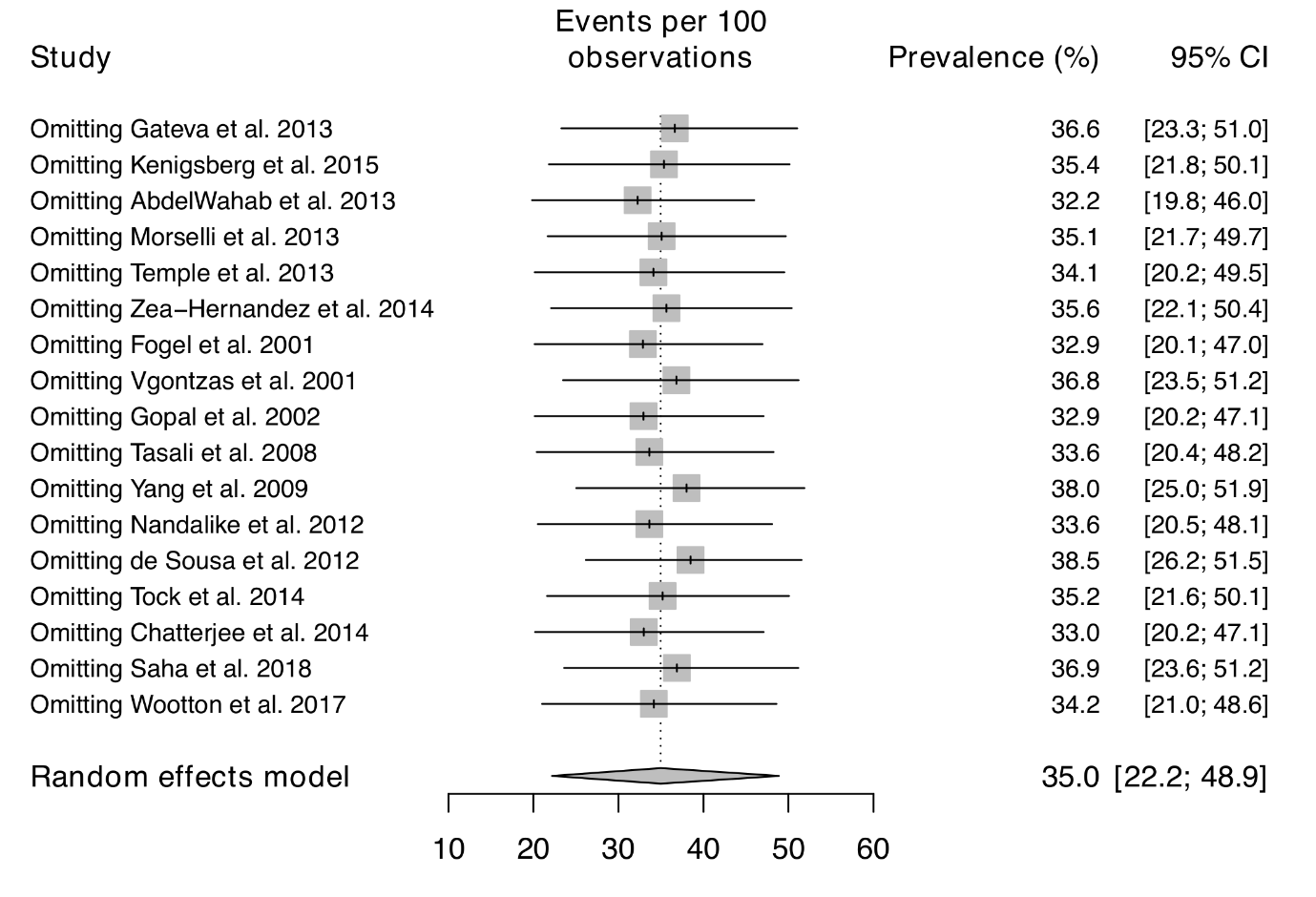


**Table S2 Factors associated with obstructive sleep apnoea (OSA) prevalence estimates identified by meta-regression analysis.**

| **Factor** | **OR (95% CI)** | **p-value** | **Explained variation (%)** |
| --- | --- | --- | --- |
| Journal article (*vs.* conference abstract) | 1.96 (0.56 to 6.80) | 0.266 | 2.0 |
| Recent (2010-2015) [*vs.* earlier (2001-2009) studies] | 0.63 (0.15 to 2.70) | 0.509 | 0.0 |
| USA (*vs.* other countries) | 1.26 (0.31 to 5.08) | 0.725 | 0.0 |
| Sample Size: 25+ (*vs.* size <25) | 0.45 (0.13 to 1.57) | 0.190 | 10.2 |
| Population |  |  | 9.4 |
| Adolescents | 1 (reference) |  |  |
| Adults | 1.29 (0.26 to 6.40) | 0.731 |  |
| Mixed | 0.38 (0.05 to 2.71) | 0.304 |  |
| PCOS Definition |  |  | 0.0 |
| NIH definition | 1 (reference) |  |  |
| Rotterdam criteria | 0.82 (0.13 to 5.27) | 0.818 |  |
| Not reported | 1.06 (0.16 to 6.88) | 0.949 |  |

**Figure S3 Schematic presentation of potential mechanisms underlying the interactions between polycystic ovary syndrome (PCOS) and obstructive sleep apnoea (OSA).**

**
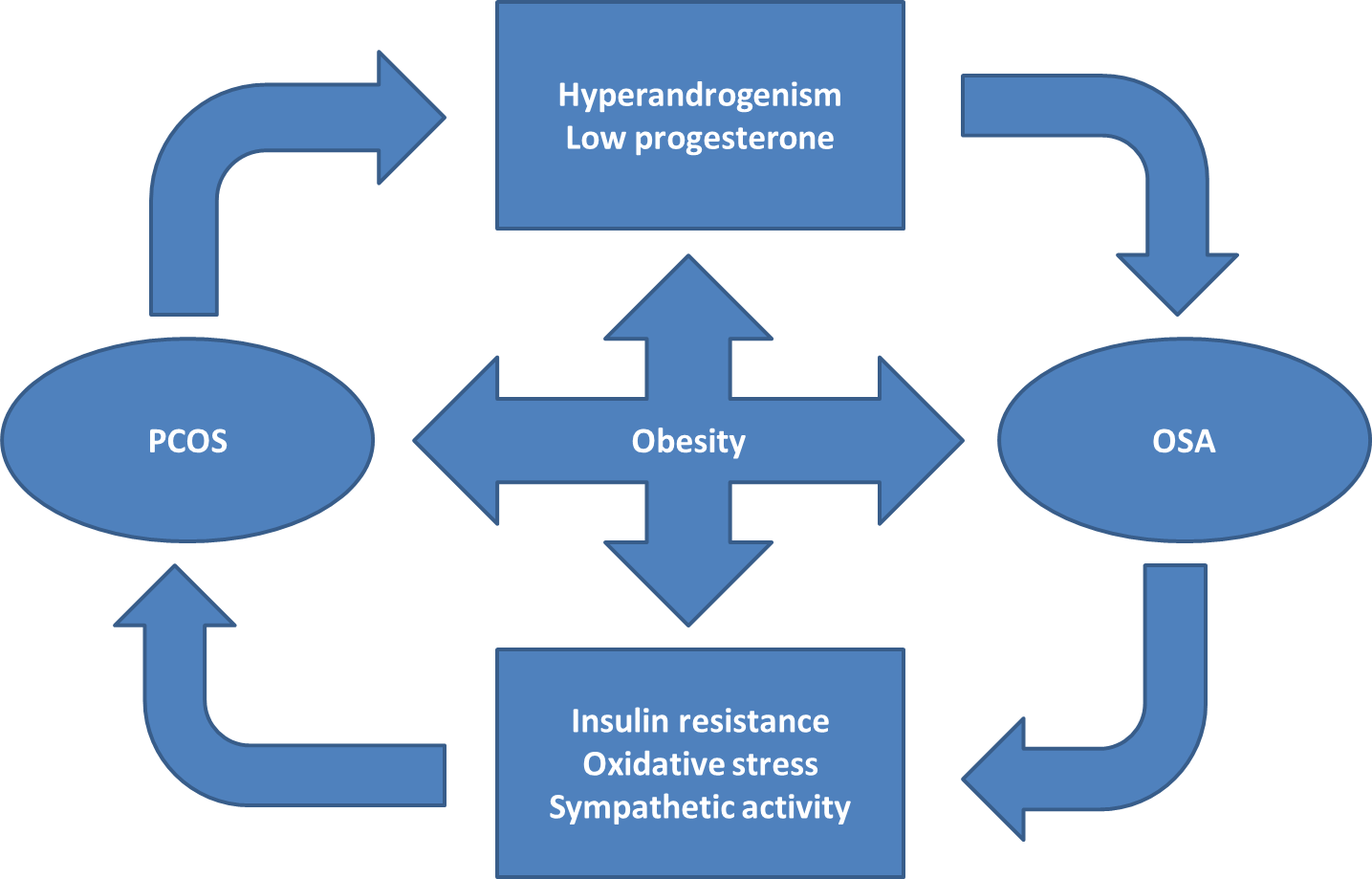
**

Polycystic ovary syndrome (PCOS) is associated with obesity, hyperandrogenism and low progesterone levels which may play a role in the development of obstructive sleep apnoea (OSA). OSA is associated with obesity, insulin resistance, oxidative stress, and increased sympathetic activity which may contribute to the pathogenesis of PCOS and also worsen its symptoms. Thus, a vicious cycle may form between these two common conditions in women.

**Appendix 1 Search strategy**

An initial search was performed on the 11^th^ of April 2016; a second search was performed on the 7^th^ of February 2017, and a third search was performed on 11^th^ of January 2019. In addition to the electronic databases below we manually searched the references of relevant papers and review articles.

- **Medline**

| **#** | **Query** | **Limiters/Expanders** |
| --- | --- | --- |
| S1 | MH "Polycystic Ovary Syndrome" | Search modes - Boolean/Phrase |
| S2 | (polycystic N3 ovar*) or PCOS or "stein leventhal" or (sclerocystic N3 ovar*) or "hyperandrogenic anovulation" | Search modes - Boolean/Phrase |
| S3 | (MH "Sleep Apnea Syndromes+") | Search modes - Boolean/Phrase |
| S4 | (sleep* N3 (apnea* or apnoea* or respirat* or breath*)) or OSA or SHS or OSAHS or SAHS or hypopnea* or hypopnoea* | Search modes - Boolean/Phrase |
| S5 | ( S1 OR S2 ) AND ( S3 OR S4 ) | Search modes - Boolean/Phrase |

- **Embase**

| **#** | **Search Terms** |
| --- | --- |
| 1 | exp ovary polycystic disease |
| 2 | ((polycystic adj3 ovar*) or PCOS or "stein leventhal" or (sclerocystic adj3 ovar*) or "hyperandrogenic anovulation").mp. [mp=title, abstract, heading word, drug trade name, original title, device manufacturer, drug manufacturer, device trade name, keyword] |
| 3 | exp sleep disordered breathing |
| 4 | ((sleep* adj3 (apnea* or apnoea* or respirat* or breath*)) or OSA or SHS or OSAHS or SAHS or hypopnea* or hypopnoea*).mp. [mp=title, abstract, heading word, drug trade name, original title, device manufacturer, drug manufacturer, device trade name, keyword] |
| 5 | 1 or 2 |
| 6 | 3 or 4 |
| 7 | 5 and 6 |

- **CINAHL (Ebsco)**

| [**#**](javascript:__doPostBack('ctl00$ctl00$FindField$FindField$historyControl$ReorderHistoryLink','')) | **Search Terms** | **Search Options** |
| --- | --- | --- |
| S1 | (MH "Polycystic Ovary Syndrome") | Search modes - Boolean/Phrase |
| S2 | (polycystic N3 ovar*) or PCOS or "stein leventhal" or (sclerocystic N3 ovar*) or "hyperandrogenic anovulation" | Search modes - Boolean/Phrase |
| S3 | (MH "Sleep Apnea Syndromes+") | Search modes - Boolean/Phrase |
| S4 | (sleep* N3 (apnea* or apnoea* or respirat* or breath*)) or OSA or SHS or OSAHS or SAHS or hypopnea* or hypopnoea* | Search modes - Boolean/Phrase |
| S5 | S1 OR S2 | Search modes - Boolean/Phrase |
| S6 | S3 OR S4 | Search modes - Boolean/Phrase |
| S7 | S5 AND S6 | Search modes - Boolean/Phrase |

- **Opengrey** **(**[**http://www.opengrey.eu/**](http://www.opengrey.eu/)**)**

((polycystic NEAR/3 ovar*) OR PCOS OR "stein leventhal" OR (sclerocystic NEAR/3 ovar*) OR "hyperandrogenic anovulation") AND ((sleep NEAR/3 (apnea* or apnoea* or respirat* or breath*)) OR OSA OR SHS OR OSAHS OR SAHS OR hypopnea* or hypopnoea*).

- **Web of Science**

| **#** | **Search Term** |
| --- | --- |
| #1 | **TOPIC:** ("polycystic ovary syndrome" or "ovary polycystic disease*" or pcos or "stein leventhal" or "hyperandrogenic anovulation") *OR* **TOPIC:** (polycystic near/3 ovar*) *OR* **TOPIC:** (sclerocystic near/3 ovar*)  *DocType=All document types; Language=All languages;* |
| #2 | **TOPIC:** ("sleep apnea syndrome" or "sleep disordered breathing") *OR* **TOPIC:** (sleep near/3 (apnea or apnoea or respirat* or breath*)) *OR* **TOPIC:** (osa or shs or osahs or sahs or hypopnea* or hypopnoea*)  *DocType=All document types; Language=All languages;* |
| #3 | #2 AND #1  *DocType=All document types; Language=All languages;* |

- **Scopus**

( TITLE-ABS-KEY ( sclerocystic W/3 ovar* ) OR TITLE-ABS-KEY ( polycystic W/3 ovar* ) OR TITLE-ABS-KEY ( "polycystic ovary syndrome" OR "ovary polycystic disease*" OR pcos OR "stein leventhal" OR "hyperandrogenic anovulation" ) ) AND ( ( TITLE-ABS-KEY ( "sleep apnea syndrome" OR "sleep disordered breathing" ) OR TITLE-ABS-KEY ( sleep W/3 ( apnea OR apnoea OR respirat* OR breath* ) ) OR TITLE-ABS-KEY ( osa OR shs OR osahs OR sahs OR hypopnea* OR hypopnoea* ) ) )

- **PsycInfo (ProQuest)**

(SU.EXACT.EXPLODE("Sleep Apnea") OR ((sleep* N/3 (apnea* or apnoea* or respirat* or breath*)) or OSA or SHS or OSAHS or SAHS or hypopnea* or hypopnoea*)) AND ((polycystic N/3 ovar*) or PCOS or "stein leventhal" or (sclerocystic N/3 ovar*) or "hyperandrogenic anovulation").

- **Endocrine abstracts (**<http://www.endocrine-abstracts.org/>**)**

| **Search terms** | **Advanced search options** |
| --- | --- |
| Polycystic ovaries and sleep apnea | Boolean  Search for grammatical variations  Similarity to search phrase: Generous differences |

- **American Endocrine Society meeting abstracts (**<http://press.endocrine.org/series/endo-meetings>**)**

Search terms: Polycystic ovary and sleep apnea

Search filters: Meeting Abstracts.

- **American Thoracic Society meeting abstracts (**<http://www.atsjournals.org/search/advanced>**)**

Search Terms: PCOS or polycystic.

- **Sleep (the joint meeting of the** [**American Academy of Sleep Medicine**](http://www.aasmnet.org/) **and the** [**Sleep Research Society**](http://www.sleepresearchsociety.org/)**) (**<http://www.sleepmeeting.org/abstract-supplements>**)**

Search terms: PCOS or polycystic.
